# Pericardial Effusions After the Arterial Switch Operation: A PHIS Database Review

**DOI:** 10.1177/21501351221146153

**Published:** 2023-03-08

**Authors:** Matthew F Mikulski, Andrew Well, Sujata Subramanian, Kathleen Colman, Charles D Fraser, Carlos M Mery, Richard P Lion

**Affiliations:** 1 Department of Surgery and Perioperative Care, 377659 Dell Medical School, The University of Texas at Austin, Austin, TX, USA; 2 Texas Center for Pediatric and Congenital Heart Disease, UT Health Austin/Dell Children's Medical Center, Austin, TX, USA; 3 Department of Pediatrics, 377659 Dell Medical School, The University of Texas at Austin, Austin, TX, USA

**Keywords:** pericardium, arterial switch operation, outcomes (includes mortality, morbidity), database (all types)

## Abstract

**Background:** Pericardial effusion (PCE) is a significant complication after pediatric cardiac surgery. This study investigates PCE development after the arterial switch operation (ASO) and its short-term and longitudinal impacts. **Methods:** A retrospective review of the Pediatric Health Information System database. Patients with dextro-transposition of the great arteries who underwent ASO from January 1, 2004, to March 31, 2022, were identified. Patients with and without PCE were analyzed with descriptive, univariate, and multivariable regression statistics. **Results:** There were 4896 patients identified with 300 (6.1%) diagnosed with PCE. Thirty-five (11.7%) with PCE underwent pericardiocentesis. There were no differences in background demographics or concomitant procedures between those who developed PCE and those who did not. Patients who developed PCE more frequently had acute renal failure (N = 56 (18.7%) vs N = 603(13.1%), *P* = .006), pleural effusions (N = 46 (15.3%) vs N = 441 (9.6%), *P* = .001), mechanical circulatory support (N = 26 (8.7%) vs N = 199 (4.3%), *P* < .001), and had longer postoperative length of stay (15 [11-24.5] vs 13 [IQR: 9-20] days). After adjustment for additional factors, pleural effusions (OR = 1.7 [95% CI: 1.2-2.4]), and mechanical circulatory support (OR = 1.81 [95% CI: 1.15-2.85]) conferred higher odds of PCE. There were 2298 total readmissions, of which 46 (2%) had PCE, with no difference in median readmission rate for patients diagnosed with PCE at index hospitalization (median 0 [IQR: 0-1] vs 0 [IQR: 0-0], *P* = .208). **Conclusions:** PCE occurred after 6.1% of ASO and was associated with pleural effusions and mechanical circulatory support. PCE is associated with morbidity and prolonged length of stay; however, there was no association with in-hospital mortality or readmissions.

## Introduction

Pericardial effusion (PCE) after pediatric cardiac surgery can be a significant complication, with severe presentations resulting in hemodynamic compromise due to cardiac tamponade. Additionally, PCE is a component of the “post-pericardiotomy syndrome,” believed to be an immune-mediated inflammatory process involving the pericardium.^1–4^ Despite the consequences of PCE, there is little evidence describing risk factors for development or guidance for perioperative management.^5–8^

Reported prevalence of postoperative PCE ranges from 1.1% to 65%, with cardiopulmonary bypass time, atrial septal defect (ASD) repair, genetic abnormalities, and first-time cardiac surgery having been identified as independent risk factors.^9–14^ However, these published studies are limited to mostly single-center reports encompassing various congenital heart disease (CHD) subtypes and surgeries, making the meaningful extrapolation to individual populations difficult. Additionally, hindering PCE research is the lack of standardized approach to identification or severity classification.^10,15^

This study sought to utilize the Pediatric Health Information System (PHIS) database to report the incidence of PCE after the arterial switch operation (ASO) for dextro-transposition of the great arteries (d-TGA) and assess for risk factors that may predispose to postoperative PCE. We chose this specific population as patients undergoing ASO have a relatively homogeneous perioperative course and would thus have similar predisposing factors and etiologies of PCE development.^16–18^ Second, the ASO cohort is easily identified with International Classification of Diseases diagnosis and procedure codes (unlike many other congenital heart surgeries^19^), mitigating concern for overlap with other pathologies. Lastly, we felt PHIS was an adequate database to answer these questions and identify ASO patients since a recent study demonstrated a 98% sensitivity for identification of ASO within the PHIS database compared to the Society of Thoracic Surgeons Congenital Heart Surgery Database.^20^

## Patients and Methods

Approval for this study was obtained after review by the Institutional Review Board for the Dell Medical School at The University of Texas at Austin (STUDY00001853, approved 10/11/2021).

### Data Source

This is a retrospective review of the PHIS database, an administrative database operated by the Children's Hospital Association (CHA), a business alliance of international children's hospitals, from January 1, 2004, to March 31, 2022.^21^ Forty-six, tertiary pediatric hospitals affiliated with the CHA submit data to PHIS containing inpatient, emergency department, observation status, and ambulatory surgery data.^22^ Data quality and reliability are assured through joint effort and agreements between participating hospitals and CHA. Data are de-identified at the time of data submission, and data are subjected to several reliability and validity checks before being included in the database. Individual patients are given a unique identifier at a hospital that carries over to multiple episodes of care at that facility, but it does not carry over to partner hospitals in the CHA. Therefore, a patient can be followed longitudinally for multiple episodes of care, provided they go to the same facility. PHIS provides an admitting diagnosis, principal diagnosis, and up to 41 additional diagnoses, in addition to a principal procedure and up to 41 additional procedures for each episode of care. From 2004 through the third quarter of 2015, diagnoses and procedures were coded using the standard *ICD, Ninth Revision* (ICD-9). Records from the fourth quarter of 2015 through the present were coded using the *Tenth Revision* (ICD-10).

### Study Population

Patients were identified by the presence of a diagnosis of d-TGA via ICD codes (745.10, 745.19, and Q20.3). Identification of ASO was additionally determined by ICD code using previously published parameters (Table S1).^19^ Demographics collected included age, sex, race, ethnicity, gestational age, and insurance status. Insurance was grouped into Private, Government (Medicare, Medicaid, Tricare, etc), and Other (eg, charity, self-pay, unknown). Clinical outcomes provided by PHIS included survival to discharge and length of stay (LOS).

PCE was defined in our cohort as those with a diagnosis code for pericardial effusion (423.9, I31.3), hemopericardium (423.0, I31.2), cardiac tamponade (423.3, I31.4), and/or those with a procedure code for pericardiocentesis or percutaneous drainage of the pericardium/mediastinum (37.0, 0W9C30Z, 0W9C3ZX, 0W9C3ZZ, 0W9C40Z, 0W9C4ZX, W9C4ZZ, 0W9D00Z, 0W9D30Z, 0W9D3ZX, 0W9D3ZZ, 0W9D40Z, 0W9D4ZX, 0W9D4ZZ) during the index hospitalization as the ASO. Further diagnoses, procedures, and outcomes were identified using ICD codes (Table S1).

Follow-up through PHIS included in the analysis was 2-fold: (1) the number of inpatient readmissions at the same index hospital and (2) the total number of hospital encounters (which encompassed all inpatient readmissions, emergency department visits, ambulatory surgery encounters, and observation status admissions) at the same hospital as the index operation throughout the study period.

### Statistical Analysis

Descriptive statistics were used for demographics, clinical characteristics, and outcomes. Categorical variables are reported as N (%). LOS is reported in median [interquartile range (IQR)] days. Chi-square and Fisher exact test were utilized to analyze noncontinuous variables. Wilcoxon signed-rank test and Kruskal-Wallis tests were used for non-normally distributed LOS comparisons between groups as indicated. All covariates that were considered clinically significant were found to be statistically significant on univariate analysis, or previously reported as significant in the literature were included in multivariable logistic and linear regressions. All variables included were assessed for collinearity. The regressions were structured such that there were 10 or more outcomes per one factor included into the model to reduce the risk of overfitting. All statistical tests were two-tailed and a *P*-value <.05 was considered significant. All statistical analyzes were performed using R and RStudio.^23^

## Results

### Study Population and Demographics

From January 1, 2004, through March 31, 2022, there were 4896 patients with d-TGA who underwent ASO. Of this cohort, 1541 (31.5%) were female, 863 (17.6%) of Hispanic background, 2297 (46.9%) had private insurance, and median gestational age was 39 [IQR: 38-39] weeks (Table 1). There were 300 (6.1%) patients with PCE at the index hospitalization, 35 (11.7%) of whom underwent pericardiocentesis, and 94 (31.3%) developed cardiac tamponade (Table 2). Pericardiocentesis occurred on median post-ASO day 5 [IQR: 1-8].

**Table 1.** Characteristics of Patients Undergoing Arterial Switch Operation With and Without Development of Pericardial Effusion.

	Total, N (%)	PCE, N (%)	No PCE, N (%)	*P* value
Total	4896	300 (6.1%)	4596 (93.9%)	–
Background demographics				
** **Female	1541 (31.5%)	85 (28.3%)	1456 (31.7%)	.227
** **White	3107 (63.5%)	198 (66%)	2909 (63.3%)	.346
** **Ethnicity				
** **Hispanic	863 (17.6%)	53 (17.7%)	810 (17.6%)	.93
** **Non-Hispanic	3222 (65.8%)	200 (66.7%)	3022 (65.8%)	
** **Unknown	811 (16.6%)	47 (15.7%)	764 (16.6%)	
** **Insurance				
** **Private	2297 (46.9%)	138 (46%)	2159 (47%)	.789
** **Government	2368 (48.4%)	150 (50%)	2218 (48.3%)	
** **Other	231 (4.7%)	12 (4%)	219 (4.8%)	
** **Median gestational age at birth (weeks) [IQR]	39 [38-39]	39 [38-39]	39 [38-39]	.475
** **History of low birth weight	268 (5.5%)	14 (4.7%)	254 (5.5%)	.526
** **History of prematurity	467 (9.5%)	31 (10.3%)	436 (9.5%)	.629
Procedural considerations				
** **Median preoperative LOS (days) [IQR]	5 [2-7]	5 [2-8]	5 [2-7]	.401
** **Balloon atrial septostomy	2287 (46.7%)	137 (45.7%)	2150 (46.8%)	.708
** **Atrial septal defect repair	4032 (82.4%)	247 (82.3%)	3785 (82.4%)	.993
** **Ventricular septal defect repair	1281 (26.2%)	78 (26%)	1203 (26.2%)	.947
** **Delayed sternal closure	431 (8.8%)	28 (9.3%)	403 (8.8%)	.738
Outcomes				
** **Acute renal failure	658 (13.4%)	56 (18.7%)	602 (13.1%)	**.006**
** **Pleural effusion	487 (9.9%)	46 (15.3%)	441 (9.6%)	**.001**
** **Blood transfusion exposure	2133 (43.6%)	137 (45.7%)	1996 (43.4%)	.449
** **Mechanical circulatory support	225 (4.6%)	26 (8.7%)	199 (4.3%)	**<.001**
** **Mechanical ventilation >96 h	1967 (40.2%)	122 (40.7%)	1845 (40.1%)	.858
** **Cardiomyopathy	2 (0%)	0 (0%)	2 (0%)	–
** **Cardiac arrest	48 (1%)	6 (2%)	42 (0.9%)	.064
Disposition and Follow-up				
** **Mortality	87 (1.8%)	10 (3.3%)	77 (1.7%)	**.035**
** **Median postoperative LOS (days) [IQR]	13 [9-20]	15 [11-24.5]	13 [9-20]	**<.001**
** **Median number of follow-up encounters [IQR]	1 [0-2]	0 [0-2]	1 [0-2]	.861
** **Median number of inpatient readmissions [IQR]	0 [0-0]	0 [0-1]	0 [0-0]	.208

Abbreviations: IQR, interquartile range; LOS, length of stay; PCE, pericardial effusion.

Bold values denote statistical significance.

**Table 2.** Pericardial Effusion Components.

Type of pericardial effusion	N	%
Total	300	–
Pericardial effusion	207	69
Hemopericardium	14	4.7
Cardiac tamponade	94	31.3
Pericardiocentesis or percutaneous pericardial drain placement	35	11.7

### Demographics

There were no associations between patient demographics or insurance status with pericardial effusions on univariate analysis (Table 1). Preoperative clinical characteristics were similar between those with and without PCE, including median gestational age at birth (39 [IQR: 38-39] vs 39 [IQR: 38-39] weeks, *P* = .475), diagnoses of low birth weight (N = 14, 4.7% vs N = 254, 5.5%, *P* = .526), and prematurity (N = 31, 10.3% vs N = 436, 9.5%, *P* = .629). After adjustment for potentially confounding factors with multivariable logistic regression, no demographic factors were associated with the development of PCE (Table 3).

**Table 3.** Multivariable Logistic Regression for Development of Pericardial Effusion.^a^

Factor	Odds ratio	95% confidence interval	*P* value
Preoperative factors			
Female	0.83	(0.63-1.07)	.154
White	1.16	(0.88-1.54)	.3
Ethnicity			
Hispanic	Reference^b^	Reference^b^	–
Non-Hispanic	0.95	(0.67-1.35)	.787
Unknown	1.31	(0.82-2.11)	.259
Low birth weight	0.83	(0.47-1.45)	.512
Procedural factors			
Balloon atrial septostomy	0.94	(0.73-1.2)	.613
Atrial septal defect repair	0.98	(0.71-1.37)	.916
Ventricular septal defect repair	0.98	(0.74-1.3)	.895
Postoperative complications			
Acute renal failure	1.4	(0.99-1.97)	.058
Pleural effusion	1.7	(1.2-2.4)	**.003**
Blood transfusion exposure	1.02	(0.77-1.34)	.914
Mechanical circulatory support	1.81	(1.15-2.85)	**.011**

^a^ Additionally adjusted for Operating Hospital and Year of operation.

^b^ Reference Category.

Bold values denote statistical significance.

### Perioperative Considerations

**Figure 1.**
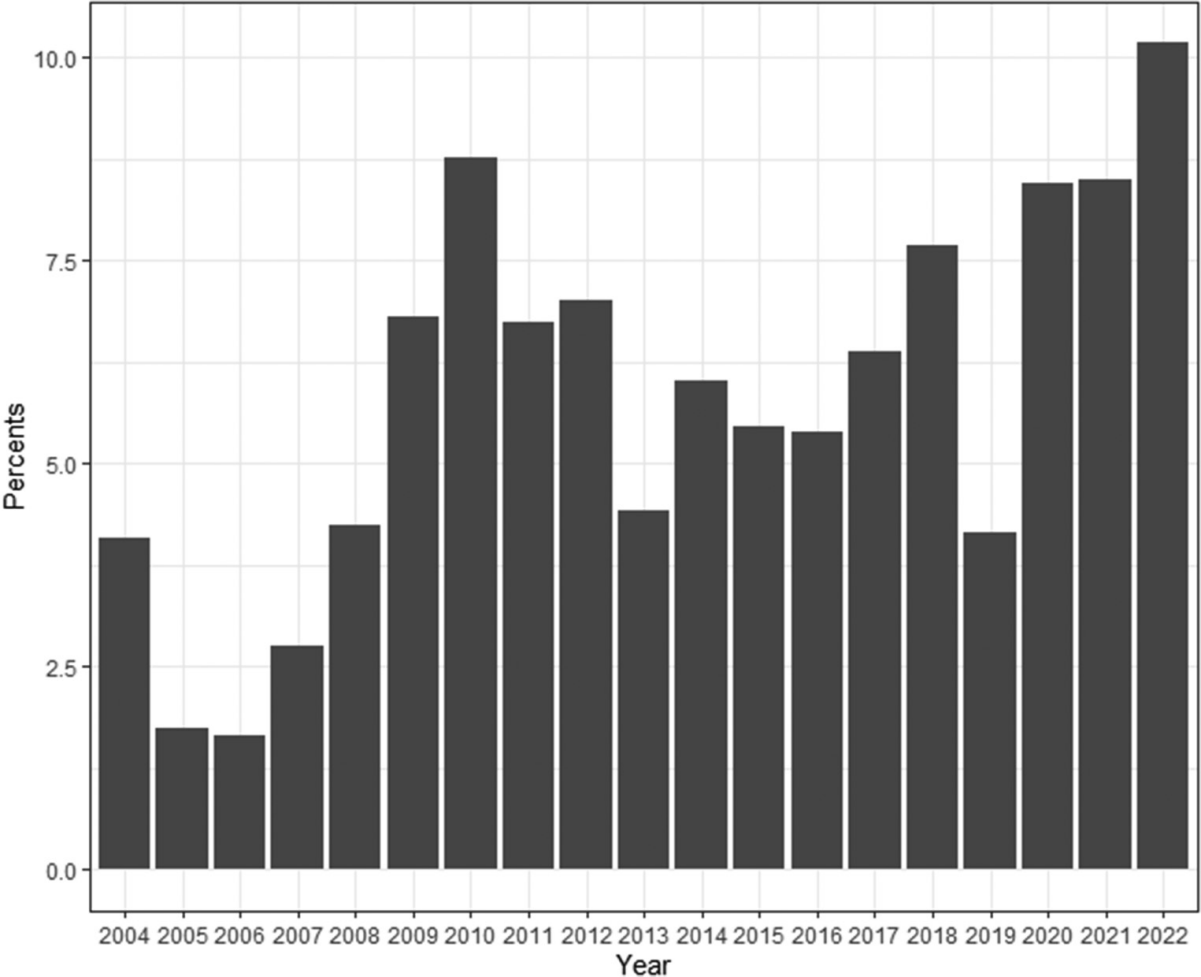
Percent incidence of pericardial effusions of all arterial switch operation by year.

There was no significant difference in perioperative factors between those who developed PCE and those who did not, including the median preoperative LOS (5 [IQR:2-8] vs 5 [IQR:2-7] days, *P* = .401) and those who underwent a balloon atrial septostomy (BAS) prior to ASO (N = 137, 45.7% vs N = 2150, 46.8%, *P* = .708). Similarly, concomitant intraoperative procedures such as ASD repair (N = 247, 82.3% vs N = 3785, 82.4%, *P* = .993) or ventricular septal defect (VSD) repair (N = 78, 26% vs N = 1203, 26.2%, *P* = .947) displayed no association with the development of PCE. After adjustment, procedural factors had no associations with the development of PCE (Table 3). The percent incidence of PCE among all undergoing ASO by year is depicted in Figure 1 with a gradual increase in PCE incidence over the duration of the study period.

### Clinical Associations

There were multiple clinical diagnoses associated with PCE (Table 1). These included a higher prevalence of acute renal failure (ARF) (N = 56, 18.7% vs N = 602, 13.1%), *P* = .006) and pleural effusions (N = 46, 15.3% vs N = 441, 9.6%, *P* = .001) compared to those without PCE. There was a significant association between those with PCE who had mechanical circulatory support (MCS) (N = 26, 8.7% vs N = 199, 4.3%, *P* < .001) compared to those without PCE during their hospitalization. After adjustment, pleural effusions had 1.7 odds (95% CI: 1.2-2.4, *P* = .003), and MCS had 1.81 odds (95% CI: 1.15-2.85, *P* = .011) of PCE (Table 3), while ARF no longer displayed an association with PCE.

### In-Hospital Mortality and Length of Stay

There were 87 (1.8%) in-hospital mortalities during the index admission. Those with a PCE had a higher rate of in-hospital mortality (N = 10, 3.3% vs N = 77, 1.7%, *P* = .035) and had a longer median postoperative LOS (15 [IQR: 11-24.5] days vs 13 [9-20] days, *P* < .001) compared to those without PCE. After adjustment, patients had a 13.34% (95% CI: 6.27%-20.88%) longer postoperative LOS if a patient had PCE (Table 4); however, PCE no longer displayed an association with in-hospital mortality (odds ratio 0.85 (95% CI: 0.33-2.14), *P* = .723) (Table 5).

**Table 4.** Multivariable Linear Regression of Postoperative Length of Stay.^a^

Factor	Percent difference	95% CI	*P* value
Preoperative factors			
Female	−3.04	(−6.2-0.22)	.068
White	−10.16	(−13.35- −6.86)	**<.001**
Ethnicity			
Hispanic	Reference^b^	Reference^b^	
Non-Hispanic	−3.79	(−8.02-0.64)	.092
Unknown	−9.41	(−14.71- −3.79)	**.001**
Low birth weight	59.34	(48.92-70.5)	**<.001**
Procedural factors			
Balloon atrial septostomy	4.46	(1.15-7.88)	**.008**
Atrial septal defect repair	−1.65	(−5.7-2.58)	.44
Ventricular septal defect repair	11.76	(7.84-15.83)	**<.001**
Perioperative factors			
Pericardial effusion	13.34	(6.27-20.88)	**<.001**
Acute renal failure	40.75	(33.82-48.04)	**<.001**
Pleural effusion	32.39	(25.63-39.53)	**<.001**
Mechanical circulatory support	104.97	(88.65-122.69)	**<.001**

^a^ Additionally adjusted for operating hospital, year of operation, and in-hospital mortalities.

^b^ Reference Category.

Bold values denote statistical significance.

**Table 5.** Multivariable Logistic Regression for In-hospital Mortality.^a^

Factor	Odds ratio	95% CI	*P* value
Preoperative factors			
Low birth weight	5.51	(2.41-12.6)	**<.001**
Procedural factors			
Ventricular septal defect repair	2.07	(1.15-3.72)	**.015**
Perioperative factors			
Pericardial effusion	0.85	(0.33-2.14)	.723
Acute renal failure	7.91	(4.17-14.99)	**<.001**
Pleural effusion	0.95	(0.41-2.16)	.898
Mechanical circulatory support	104.15	(51.79-209.42)	**<.001**

^a^ Additionally adjusted for operating hospital and year of operation.

Bold values denote statistical significance.

### Follow-up

There were 2298 total readmissions at the index hospital involving 1150 (23.5%) patients. Of these readmissions, 46 (2%) involved any diagnosis of PCE and 9 (0.4%) underwent pericardiocentesis. There was no difference in the median number of readmissions between those with and without a diagnosis of PCE that occurred during the index hospitalization for ASO (Table 1). Additionally, there was no difference in the total number of subsequent all-encompassing hospital encounters at the index institution between those with and without PCE during the index hospitalization.

## Comment

This study reports an overall prevalence of PCE after ASO of 6.1% after reviewing 4896 patient cases from the PHIS database over 18 years (Table 1). This is similar to previous reports of PCE after ASO ranging from 0.0% to 6.7%.^9,11,12^ There were multiple morbidities associated with PCE, including pleural effusion and MCS, despite not knowing the exact temporal associations with PCE. Although there was no association with mortality, PCE was associated with a longer postoperative LOS.

### Demographics and Perioperative Characteristics

This study did not identify any association of PCE with background demographics or birth characteristics. It is known that there is a strong male preponderance for d-TGA (60%-70%),^11,17,24^ but this study did not find any associations between sex and PCE. This stands in contrast to that found in Cheung and colleagues’ review of 77 PCE patients with all-encompassing CHD diagnoses in which they found female sex to significantly increase PCE with a relative risk of 2.05, although their study is limited by a small sample size and could be influenced by sex differences in underlying CHD diagnoses and surgeries performed.^11^ Of note, the cohort included 15 ASO patients with similar male predominance (73.3%) and only 1 reported PCE.^11^

The lack of associations with baseline patient characteristics of this relatively homogeneous patient population may suggest intraoperative or postoperative factors as reasons for the development of PCE.^16,17^ When looking at procedural considerations, however, BAS, ASD repair, and VSD repair were not associated with the development of PCE. Because of the refinement in surgical technique across institutions and over time, and with the trend of increasing percent incidence of PCE over the study period, operating hospital and discharge year were incorporated into our regression models. However, our analysis was limited by the inability to look at individual intraoperative technical and procedural components such as surgical approach, anesthesia duration, cardiopulmonary bypass strategy, and mediastinal chest tube management.^9,10^

### Fluid Overload and Inflammatory State

The main associations identified in this study include the development of PCE in the setting of pleural effusion (∼15%) and MCS (∼9%), even when adjusting for preoperative and intraoperative factors, operating center, and year of procedure. MCS demonstrated the highest odds of PCE (1.81). Within the limitations of analyzing an administrative database—where we cannot elucidate the timing of each diagnosis—this suggests that fluid overload and a systemic inflammatory state may influence the development of PCE and should be a focus of future research.^25^

### Hospital Course and Survival to Discharge

This study identified an overall in-hospital mortality rate among those undergoing ASO at 1.8%—in keeping with previous reports.^17,26^ The rate of in-hospital mortality was twice as high if a patient had a PCE; however, this association did not persist on multivariable analysis. The associations found likely reflected the complexity of disease. For example, the addition of a VSD repair increases a patient's Society of Thoracic Surgeons-European Association for Cardio-Thoracic Surgery mortality score for ASO, low birth weight patients can be technically more difficult, and MCS indicates a cardiac or respiratory failure with a need for increased support. For postoperative LOS, this study did identify on both univariate and multivariable analysis that PCE was associated with prolonged LOS. These together reinforce that PCE after pediatric cardiac surgery can have significant clinical consequences, demand increased resource utilization, and highlight the need to identify high-risk patients and implement effective screening and treatment when appropriate.

### Long-Term Follow-up

After reviewing more than 140 000 pediatric cardiac surgical cases, Elias et al reported only a 1.1% overall readmission rate for PCE after congenital heart surgery.^7^ Interestingly, they report more complex surgeries such as ASO conferred less risk of readmission compared to isolated ASD repairs.^7^ However, they did not report the number of patients who developed PCE during their initial postoperative hospitalization. This study found a similar rate of readmission for PCE after the ASO (2%), although the presence of PCE during index hospitalization did not result in increased readmissions or overall hospital encounters compared to those without PCE.

### Limitations

This study represents an analysis of PCE after ASO using a large, longitudinal database with multiple validity checks on data included. However, there are several limitations inherent in the use of an administrative dataset reliant on correct documentation of patient diagnostic and procedural billing codes. Using ICD-9/10, there is no specific procedure code for ASO. The authors relied on other specific procedure and diagnosis combinations to exclude other surgeries,^19^ with reported results likely a conservative estimate of patients undergoing ASO. Additional perioperative variables of interest, such as cardiopulmonary bypass time, are not collected by PHIS. Perioperative medication usage, specifically NSAIDs or nephrotoxic agents that could contribute to ARF, was not analyzed as part of this study. Future studies should include awareness of medication regimen as it has been suggested that empiric aspirin utilization confers a lower risk of PCE in adults after cardiac surgery.^6^

Although it is unlikely that a patient undergoing ASO had the procedure at another hospital, it is possible that there are duplicated patients in the cohort if they move from one hospital to another. Additionally, while PHIS tracks longitudinal hospital encounters at the same institution, this study likely does not identify all follow-up encounters since they may present to another hospital for subsequent care.^7^

## Conclusions

PCE after ASO is a frequent occurrence with a prevalence of 6.1% and is associated with increased morbidity, including a prolonged postoperative length of stay. However, there was no association with in-hospital mortality or readmissions. Pleural effusion or undergoing temporary MCS was identified as independent risk factors in the development of PCE.

## Supplemental Material

sj-docx-1-pch-10.1177_21501351221146153 - Supplemental material for Pericardial Effusions After the Arterial Switch Operation: A PHIS Database Review Click here for additional data file. Supplemental material, sj-docx-1-pch-10.1177_21501351221146153 for Pericardial Effusions After the Arterial Switch Operation: A PHIS Database Review by Matthew F Mikulski, Andrew Well, Sujata Subramanian, Kathleen Colman, Charles D Fraser Jr., Carlos M Mery and Richard P Lion in World Journal for Pediatric and Congenital Heart Surgery

## Abbreviations and Acronyms

ARF: acute renal failure
ASD: atrial septal defect
ASO: arterial switch operation
BAS: balloon atrial septostomy
CHA: Children's Hospital Association
CHD: congenital heart disease
d-TGA: dextro-Transposition of the Great Arteries
ICD: International Classification of Diseases
IQR: interquartile range
LOS: length of stay
MCS: mechanical circulatory support
PCE: pericardial effusion
PHIS: Pediatric Health Information System
VSD: ventricular septal defect
