# Supplementary material for: Pericardial Effusions After the Arterial Switch Operation: A PHIS Database Review
Source: World J Pediatr Congenit Heart Surg. 2023 Mar 8;14(2):148–54. doi: 10.1177/21501351221146153 (PMC10041572; doi:10.1177/21501351221146153)
Supplement: sj-docx-1-pch-10.1177_21501351221146153 - Supplemental material for Pericardial Effusions After the Arterial Switch Operation: A PHIS Database Review [file sj-docx-1-pch-10.1177_21501351221146153.docx]

**Pericardial Effusions after the Arterial Switch Operation: A PHIS Database Review**

**Table S1**

| **Diagnoses codes** | |
| --- | --- |
| VSD | 7454,Q210 |
| ASD | 7455,Q211 |
| d-TGA | 74510,74519,Q203 |
| Pericardial effusion | 4239, I313 |
| Hemopericardium | 4230, I312 |
| Cardiac tamponade | 4233, I314 |
| Acute renal failure | 5845-9, N170, N171, N172, N178, N179 |
| Cardiac arrest | 4275, I462, I468, I469, I97121, I97711 |
| Pleural effusion | 5119, J90, J918 |
| Low birth weight | 764, 7641, 7642, 7649, 76401-8, 76411-8, 76421-8, 76491-8, 765, 76501-8, 76511-8, P0501-8, P0511-8, V2130-5, P0700-3, P0710, P0714-8 |
| Prematurity | 7652, 76521-8, 3622, 36221-7, 76500-26, P0730-9, H35101-3, H35 )109, H35111-3, H35119, H35121-3, H35129, H35131-3, H35139, H35141-3, H35149, H35151-3, H35159, H35161-3, H35169 |
| **Procedure codes** | |
| Blood transfusion | 9900, 9902-4, 30233N0, 30233N1, 30233P0, 30233P1, 30233H0, 30233H1, 30243H0, 30243H1, 30243N0, 30243N1, 30243P0, 30243P1 |
| Mechanical ventilation >96 hours | 9672, 5A1955Z |
| Temporary mechanical circulatory support (including extracorporeal membranous oxygenation) | 3760, 3765, 3965, 5A02116, 5A0211D, 5A02216, 5A0221D, 02HA3RZ, 3768, 02HA0RS, 02HA3RS, 02HA4RS, 5A02116, 5A02216, 02HA0RZ, 02HA4RZ, 5A02216, 5A15223, 5A1522F, 5A1522G, 5A1522H |
| Pericardiocentesis or percutaneous drainage of the pericardium/mediastinum | 370, 0W9C30Z, 0W9C3ZX, 0W9C3ZZ, 0W9C40Z, 0W9C4ZX, W9C4ZZ, 0W9D00Z, 0W9D30Z, 0W9D3ZX, 0W9D3ZZ, 0W9D40Z, 0W9D4ZX, 0W9D4ZZ |
| Delayed sternal closure | 7841, 0PQ00ZZ, 0PQ03ZZ, 0PQ04ZZ, 0PQ0XZZ |
| Balloon atrial septostomy | 3541, 3542, 02163Z7, 02164Z7 |
| VSD repair | 3553,3562,3572, all codes including 02RM0,02UM0,02QM0  vsdrep=1 AND tofrep=0 AND avsdrep=0 AND ASO=0 AND truncusrep=0 AND coarep=0 AND iaarep=0 AND tapvrrep=0 AND shunt=0 AND norwood=0 AND glenn=0 AND fontan=0 AND arch=0 AND hlhs=0 AND avsd=0 AND TOF=0 AND TGA=0 AND truncus=0 AND pavsd=0 |
| ASD repair | 3571,3551,3561, all codes including 02R50,02Q50,02U50  asdrep=1 AND vsdrep=0 AND avsdrep=0 AND tofrep=0 AND truncusrep=0 AND coarep=0 AND iaarep=0 AND ASO=0 AND tapvrrep=0 AND shunt=0 AND norwood=0 AND glenn=0 AND fontan=0 AND avsd=0 AND arch=0 AND hlhs=0 AND pavsd=0 AND TGA=0 AND TOF=0 AND truncus=0 |
| Arterial switch operation | 3584  Coronary reposition: all codes including 02S0,02S1  Aortic reposition: all codes including 02SW,02SX  Pulmonary artery reposition: all codes including 02SP,02S1  TGA code + coronary reposition code  TGA code + aortic reposition code  TGA code + pulmonary artery reposition code  TGA=1 AND ASO=1 AND pavsd=0 AND rvpa=0 AND tofrep=0 AND shunt=0 AND avsd=0 AND avsdrep=0 AND truncus=0 AND truncusrep=0 AND hlhs=0 AND norwood=0 AND Glenn=0 AND Fontan=0 AND arch=0 AND coarep=0 AND iaarep=0 |
